# Effect of exposure-based vs traditional cognitive behavior therapy for fibromyalgia: a two-site single-blind randomized controlled trial

**DOI:** 10.1097/j.pain.0000000000003128

**Published:** 2023-12-15

**Authors:** Maria Hedman-Lagerlöf, Nils Gasslander, Alice Ahnlund Hoffmann, Maria Bragesjö, Amanda Etzell, Simon Ezra, Elsa Frostell, Erik Hedman-Lagerlöf, Caroline Ivert, Björn Liliequist, Brjánn Ljótsson, Johanna M. Hoppe, Josefin Palmgren, Edward Spansk, Felicia Sundström, Josefin Särnholm, Georgia Tzavara, Monica Buhrman, Erland Axelsson

**Affiliations:** a Centre for Psychiatry Research, Department of Clinical Neuroscience, Karolinska Institutet & Stockholm Health Care Services, Region Stockholm, Stockholm, Sweden; b Division of Clinical Psychology, Department of Psychology, Uppsala University, Uppsala, Sweden; c Division of Psychology, Department of Clinical Neuroscience, Karolinska Institutet, Stockholm, Sweden; d Gustavsberg Primary Health Care Center, Region Stockholm, Stockholm, Sweden; e Division of Family Medicine and Primary Care, Department of Neurobiology, Care Sciences and Society, Karolinska Institutet, Stockholm, Sweden; f Liljeholmen Primary Health Care Center, Region Stockholm, Stockholm, Sweden; g Academic Primary Health Care Center, Region Stockholm, Stockholm, Sweden

**Keywords:** Fibromyalgia, Randomized controlled trial, Cognitive behavior therapy, Exposure

## Abstract

Fibromyalgia is a debilitating pain condition for which treatment effects are typically modest. The most evaluated psychological treatment is traditional cognitive behavior therapy (T-CBT), but promising effects have recently been seen in exposure-based cognitive behavior therapy (Exp-CBT). We investigated whether Exp-CBT was superior to T-CBT in a randomized controlled trial. Self-referred participants with fibromyalgia (N = 274) were randomized (1:1) to 10 weeks of Exp-CBT or T-CBT. Treatments were delivered online and presented as “CBT for fibromyalgia.” Participants were assessed at baseline, weekly during treatment, posttreatment, and at 6- and 12-month follow-up. Primary outcome was the difference in reduction in fibromyalgia severity as measured using the Fibromyalgia Impact Questionnaire (FIQ) over 11 assessment points from baseline to posttreatment, modelled within an intention-to-treat framework using linear mixed effects models fitted on multiple imputed data. Approximately 91% of weekly FIQ scores were collected over the main phase. There was no significant difference between Exp-CBT and T-CBT in the mean reduction of fibromyalgia severity from pretreatment to posttreatment (b = 1.3, 95% CI −3.0 to 5.7, *P* = 0.544, d = −0.10). Minimal clinically important improvement was seen 60% in Exp-CBT vs 59% in T-CBT. Effects were sustained up to 12 months posttreatment. This well-powered randomized trial indicated that Exp-CBT was not superior to T-CBT for fibromyalgia. Both treatments were associated with a marked reduction in fibromyalgia severity, and the online treatment format might be of high clinical utility. T-CBT can still be regarded a reference standard treatment that remains clinically relevant when compared to novel treatment approaches.

## Abstract 2

Supplemental Digital Content is Available in the Text.

Although third-wave cognitive behavior therapy has grown in popularity and clinical dissemination, traditional cognitive behavior therapy is a treatment option that fares well in direct comparison to novel treatment approaches.

## 1. Introduction

Fibromyalgia is a chronic pain condition that commonly involves fatigue, impaired sleep, and cognitive disturbances.^42^ The condition has a global prevalence of approximately 2% to 4%^40^ and can be highly debilitating and difficult to treat, which results in a substantial burden on patients, health care, and society.^15^ An important pathophysiological process is believed to be that of central sensitization: the amplification of neural signaling by the central nervous system.^38^ Pharmacological treatments have shown mostly modest effects.^39^ Best practice guidelines promote the use of nonpharmacological interventions, including psychological treatments.^33^

Traditional cognitive behavior therapy (T-CBT) is the criterion standard psychological treatment for fibromyalgia, evaluated in over 40 randomized controlled trials (RCTs).^7^ Traditional cognitive behavior therapy focuses on teaching coping strategies to manage pain, with the aim of achieving beneficial effects on mood and quality of life.^14,22^ Typically, this includes cognitive restructuring techniques, stress management techniques, applied relaxation, physical exercise, and pacing: balancing activity and rest.^10,20^ Traditional cognitive behavior therapy yields small between-group effects on pain (d = −0.30), disability (d = −0.31), and negative mood (d = −0.34) as compared to mostly rudimentary control conditions, such as no treatment, treatment as usual, and placebo.^6^ More high-quality trials that compare T-CBT with other active treatments^8,36,48^ are needed to move the field forward and learn how to produce larger effects.

Several recent strains of CBT including modern exposure-based CBT (Exp-CBT)^8,16,53^ and “third-wave” treatments, such as acceptance and commitment therapy,^34^ aim to reduce behavioral avoidance, which has been found to be an important predictor of pain chronicity.^21,52,54^ Exposure-based CBT puts particular emphasis on systematic exposure, meaning that patients repeatedly and voluntary approach situations that give rise to pain and pain-related distress, to achieve beneficial long-term effects. We have developed an Exp-CBT protocol that can be delivered online with therapist guidance. In a randomized controlled trial, this treatment produced moderate to large effects on fibromyalgia severity, fatigue, disability, and quality of life in comparison to a waitlist control (d = 0.73-0.91).^19^ There was also a large between-group effect on pain intensity (d = 0.86) which is unusual. This led us to the hypothesis that Exp-CBT could be more efficacious than psychotherapies of the kind usually used for fibromyalgia (see above). However, before the present trial, Exp-CBT had not yet been directly compared with another active treatment such as the criterion standard T-CBT.

We compared the clinical efficacy of Exp-CBT with that of T-CBT for fibromyalgia in a randomized controlled trial. Based on results from earlier trials, we hypothesized that Exp-CBT would produce a larger reduction in fibromyalgia severity over the 10-week treatment period. To increase availability and ensure stringent experimental control over the treatment content, Exp-CBT and T-CBT were delivered in the same therapist-guided online format.

## 2. Methods

### 2.1. Trial oversight

This was a single-blind randomized controlled trial of internet-delivered Exp-CBT vs internet-delivered T-CBT for adults with fibromyalgia. Study sites were Karolinska Institutet, Stockholm, Sweden, and Uppsala University, Uppsala, Sweden. The trial was preregistered at ClinicalTrials.gov (NCT05058911) and approved by the Swedish Ethical Review Authority (2021-03302). All study procedures adhered to the Declaration of Helsinki and current legislation pertaining to the management of personal data. Results are reported in accordance with the Consolidated Standards for Reporting Trials (CONSORT) statement.^45^

### 2.2. Participants

To be eligible for the trial, participants were required to (1) be aged at least 18 years, (2) have been diagnosed with fibromyalgia by a physician, (3) be living in Sweden, and (4) have continuous access to a computer, tablet, or smartphone with internet access. Exclusion criteria were (1) severe symptoms of depression as indicated by ≥30 on the Montgomery–Åsberg Depression Rating Scale-Self-Rated (MADRS-S),^47^ (2) suicidal ideation as indicated by ≥4 on the suicidality item of the MADRS-S, (3) psychosis, (4) an alcohol or substance use disorder likely to severely interfere with treatment, (5) ongoing psychological treatment, (6) pregnancy beyond week 29, (7) a medical condition other than fibromyalgia requiring immediate treatment or being the primary condition, and (8) insufficient language or computer skills to take part in a text-based online treatment. Participants were allowed to use psychotropic medication if the regimen was stable for at least 4 weeks before randomization, with the intention of keeping it constant during treatment.

### 2.3. Recruitment and determination of eligibility

Participants were self-referred through the study Web site, and we advertised the trial primarily through social media, national newspapers, patient organizations, and recruitment posters. The caption of the advertisement read “Are you bothered by fibromyalgia?”. Applicants logged in to the secure study web platform using electronic identification, received written information about the trial, provided informed consent using a web form, and then completed an online screening battery of self-report questionnaires. Complete applications prompted a structured eligibility interview with a licensed psychologist or master-level psychologist student (A.A.H., M.Br., A.E., S.E., E.F., N.G., C.I., M.H-L., B.Li., J.M.H., J.P., E.S., F.S., J.S., and G.T.). All assessors received continuous support and supervision from the steering committee. Applicants who did not meet all criteria for participation were excluded and referred to routine care. Those who met all eligibility criteria were included as participants if they completed the pretreatment assessment. When we had reached the target of 270 participants, no further applicants were scheduled for an eligibility interview. Those who were already booked were assessed for eligibility, which resulted in a final sample size of 274.

### 2.4. Randomization and masking

Participants were randomized (1:1) to Exp-CBT or T-CBT in 4 consecutive even-numbered cohorts, by an individual otherwise not involved in the trial, using a true random number service (www.random.org). The individual conducting the randomization did not have access to personal or clinical information about the participants, and the randomization took place after the pretreatment assessment and inclusion of the applicant in the trial. It was therefore not possible to predict treatment allocation of future participants or to match participants to their treatment. For the entirety of the trial, we strived to keep all participants blinded to the precise nature of the 2 CBTs tested and which type of CBT that each participant had been allocated to. Thus, although the study information stated that CBT had shown promise for fibromyalgia and that 2 CBT protocols would be compared in a randomized controlled trial, there was no mention of Exp-CBT or T-CBT specifically, and both treatments were referred to simply as “CBT for fibromyalgia.” At posttreatment, we conducted a blinding check where participants were asked whether they were aware of which 2 treatments that were compared in the trial, which treatment that the researchers believed would be superior, and which of the 2 treatments that the participants themselves had been assigned to. The analyses of the most important efficacy outcomes were also conducted by an individual blinded to treatment identity (see the “Statistical analysis” section).

### 2.5. Interventions

#### 2.5.1. Online treatment format

Both treatments spanned 10 weeks and were variants of therapist-guided internet-delivered CBT which is a widely implemented and evaluated treatment format.^18^ Participants regularly logged in to the secure online treatment platform where the treatment content necessary for behavior change was conveyed primarily through an illustrated text divided into 8 interactive online modules that could also be downloaded as audio files. Each module included psychoeducational materials, homework exercises, worksheets, and questions for reflection. Each week, participants were encouraged to report back to their online therapist to proceed to the next module. Communication transpired through email-like messages, where participants could expect a response within 2 workdays. Participants were also telephoned for the purpose of problem-solving inactivity and sometimes for clarifying treatment instructions and providing technical support. The Exp-CBT and T-CBT modules were written to be equal in length and were matched for readability as indicated by a LIX readability index around 35 to 40, which indicates medium difficulty.^1^

#### 2.5.2. Therapist support and supervision

This trial was designed to ensure that both therapies were delivered by allegiant therapists, supervised by experts, and with little threat of cross-contamination. Exposure-based CBT was delivered at Karolinska Institutet, and T-CBT was delivered at Uppsala University. At each site, therapists were licensed psychologists or master-level psychologist students, and the work groups of the 2 study sites were matched for competence and previous experience (Exp-CBT: A.A.H., M.Br., E.F., C.I., B.Li., J.P., and J.S.; T-CBT: A.E., S.E., N.G., J.M.H., E.S., F.S., and G.T.). The therapists attended weekly supervision with the main author of the corresponding treatment protocol (M.H-L. and M.Bu., respectively) who strived to build allegiance and promote adherence to the corresponding treatment components. The main role of the therapist in both treatments was to support progress through positive reinforcement, to address questions that arose, and to help the participant in tailoring the treatment content including daily homework on an individual basis.

#### 2.5.3. Exposure-based cognitive behavior therapy

We administered Exp-CBT in accordance with the previously evaluated protocol.^19^ The fundamental tenet of this treatment is that behavioral avoidance and safety behaviors are likely to lead to chronicity or even deterioration in the long term. The full 10 weeks were therefore heavily focused on exposure, meaning that participants were encouraged to repeatedly and systematically engage in activities that gave rise to pain and pain-related distress (exposure) while refraining from behaviors intended to reduce pain and pain-related distress in the short term (response prevention). Although the fear of pain was the focus of some exposure exercises, this was not the sole target of exposure in this protocol. Rather, participants were encouraged to approach situations and phenomena that gave rise to unwanted physical sensations or symptom-related distress and also to abstain from behaviors contingent on distress in a wider sense. The initial phase of treatment focused on psychoeducational content, and participants were encouraged to identify and track their avoidance behaviors before proceeding to exposure exercises tailored to the individual and his or her pattern of behavioral avoidance. For instance, those who avoided physical activity due to pain or pain-related distress were encouraged to become more physically active, whereas individuals who strived to be persistently preoccupied to distract themselves from pain or pain-related distress were encouraged to abstain from this strategy and instead practice observing unwanted physical sensations. Exposure exercises were rated for expected pain and discomfort. Participants were instructed to engage with the exercises in an order they preferred and encouraged to vary between exercises rated high and low throughout treatment to maximize learning. Systematic mindfulness training was an integrated component throughout treatment, meaning that participants practiced intentionally observing and labeling symptoms and other experiences in the present moment. This was intended as a means of increasing the participant's willingness to engage in exposure. For a module-per-module overview of Exp-CBT, see Table 1 in the Supplemental digital content (available at http://links.lww.com/PAIN/B966).

#### 2.5.4. Traditional cognitive behavior therapy

We administered T-CBT in accordance with a protocol previously evaluated for chronic pain,^10^ with small changes primarily to ensure that module 1 of both treatments began with identical atheoretical texts about fibromyalgia (see Table 1, available at http://links.lww.com/PAIN/B966). The rationale for T-CBT included a broader set of potential exacerbating and maintaining factors than Exp-CBT. In T-CBT, fibromyalgia was described as involving a large number of problems such as various forms of pain and fatigue, depressed mood, anxiety, sleep disturbances, impaired social capacity, and reduced physical activity. The treatment emphasized that the individual may address any or all of these areas based on individual needs and wants, to achieve beneficial long-term effects. Week by week, the patient was presented with a wide spectrum of components, including activity planning, pacing (balancing activity and rest), relaxation, cognitive restructuring techniques, sleep-promotion techniques, stress management strategies, assertiveness training, and prescribed physical activity. The emphasis of T-CBT was on patients working continuously with activity planning and making use of strategies such as pacing and relaxation as they saw fit before, during, and after activities to manage pain and pain-related distress.

### 2.6. Outcomes

Participants completed self-report questionnaires online at screening, at pretreatment, after week 1 through 9 in treatment, at posttreatment, and at 6- and 12-month follow-up. All 6 core outcome domains of the IMMPACT recommendations were assessed.^50^ Primary outcome was the Exp-CBT vs T-CBT difference in change in fibromyalgia severity (the combination of pain, physical functioning, and emotional functioning) over the treatment period, as assessed using the Fibromyalgia Impact Questionnaire (FIQ)^11^ administered over the corresponding 11 weekly measurement points. The FIQ is a validated and disease-specific instrument known to be reliable and responsive to change.^5^ Notably, we chose the original FIQ over its revised version because this allowed us to conduct a more reliable power analysis based partially on estimates from previous trials.^12,19,23,32,43^ Pain intensity was measured using the Fibromyalgia Impact Questionnaire-Pain subscale (FIQ-pain)^11^ and the Brief Pain Inventory-Short Form, Severity subscale (BPI-SF).^35^ Fatigue was measured using the Fatigue Severity Scale (FSS).^27^ Depressive symptoms were measured using the 2-item Patient Health Questionnaire (PHQ-2),^24^ and general anxiety was measured using the 2-item Generalized Anxiety Disorder (GAD-2).^25^ Pain catastrophizing was measured using the Pain Catastrophizing Scale (PCS),^46^ and pain-related avoidance behavior was measured using the Psychological Inflexibility in Pain Scale-Avoidance subscale (PIPS-A).^56^ Disability was measured using the 12-item World Health Organization Disability Assessment Schedule 2 (WD2-12),^51^ and quality of life was measured using the Brunnsviken Brief Quality of Life (BBQ).^30^ Physical activity at baseline was measured using the Godin-Shephard Leisure-Time Physical Activity Questionnaire (GSLTPAQ). Owing to the risk that very high or very low values were not valid and comparable, we dichotomized the GSLTPAQ to indicate active (≥24) vs insufficiently active (<24) physical activity. At week 2, we administered the Credibility/Expectancy scale (C/E-scale) as a measure of treatment credibility and the participant's expectancy of improvement^9^ and a brief version of the Working Alliance Inventory (WAI) as a measure of the strength of the relationship with the therapist.^31^ Treatment satisfaction was measured using the 8-item Client Satisfaction Questionnaire (CSQ-8).^28^ As is detailed in the preregistered study protocol, a few additional scales were administered primarily for the purpose of cost-effectiveness and mediation analyses which will be reported in secondary publications.

### 2.7. Patient involvement

The Exp-CBT protocol has been continuously developed based on feedback from patients in previous trials.^19,32^ There was no direct patient involvement in the design, data collection, or analysis in the present trial. The Swedish fibromyalgia patient association was contacted and informed about the trial and assisted in the recruitment by posting information on their Web site. We thoroughly assessed patient safety aspects and the work load of participating in the study and monitored symptom measures on a weekly basis. Participants were encouraged to communicate with their therapist if any concerns regarding the participation in the trial arose. We plan to disseminate the results to study participants, healthcare professional, and patients with fibromyalgia through the Swedish fibromyalgia patient association.

### 2.8. Power analysis

Based on estimates from previous trials,^12,19,23,32,43^ we conducted an a priori power analysis based on Monte Carlo simulations (n = 1000) of linear mixed-effects regression analyses with 11 weekly assessment points, an expected Exp-CBT mean FIQ score reduction of 16.9,^19^ a T-CBT mean FIQ score reduction of no more than 9,^12,23,43^ and a data loss of 10% after baseline, finding that 130 participants would be needed per arm (Exp-CBT vs T-CBT) for 80% power in testing the time × group coefficient (α = 0.05). In early January 2022, because the initial missing data rate was higher than expected, we reconsidered the assumption of 10% data loss and posited that this could rise as high as 20% to 25%. This resulted in a revised target sample size of 135 per arm.

### 2.9. Statistical analysis

The data analysis followed a preregistered plan and was conducted in R 4.2.0^41^ with the mice 3.14.0 and nlme 3.1-157 packages. Blinding of participant condition during analyses was achieved by the use of a data set without information about treatment identity. All efficacy analyses of continuous outcomes adhered to the intention-to-treat principle, which means that all participants who were randomized and included in the trial were included in the analysis regardless of adherence or the rate of missing data. Missing values were first imputed for 20 data sets using predictive mean matching under hierarchical multiple imputation by chained equations.^55^ This was performed separately for each treatment group to maintain group-specific effects. Linear mixed-effects models that included a random intercept and slope (time) were then fitted using maximum likelihood estimation, and the multiply imputed data sets were combined using the Rubin rules. Covariance over the random effects was unstructured, and a first-order autoregressive residual covariance structure, chosen based on model fit, was used for outcomes that had been measured at more than 2 time points. Fixed effects were time point, treatment group, and the time point × group interaction; the latter of which was tested to determine the relative efficacy of Exp-CBT and T-CBT. For analyses of the follow-up period, piecewise models were fitted with a spline at the posttreatment assessment. All reported coefficients correspond to average change over the entire period of interest, either pretreatment to posttreatment or pretreatment to 12 months. Standardized effect sizes were calculated as the model-implied change, or group difference in change, divided by observed full sample standard deviation at the beginning of the corresponding period (ie, pre for both the pre to post estimates and the pre to 12-month estimates). Scores on the resulting statistic, Cohen d, are usually denoted large around ±0.8, moderate around ±0.5, and small around ±0.2.^13^ Minimal clinically important change (the smallest change of clinical relevance) was defined as a 14% change from the baseline on the FIQ; a standard criterion that has been derived from the empirical relationship of the FIQ to the Patient Global Impression of Change.^4^ Categorical and dichotomous outcomes were analyzed using the χ2 test, with effect sizes reported as risk ratios (RRs).

## 3. Results

### 3.1. Sample characteristics

**Figure 1.**
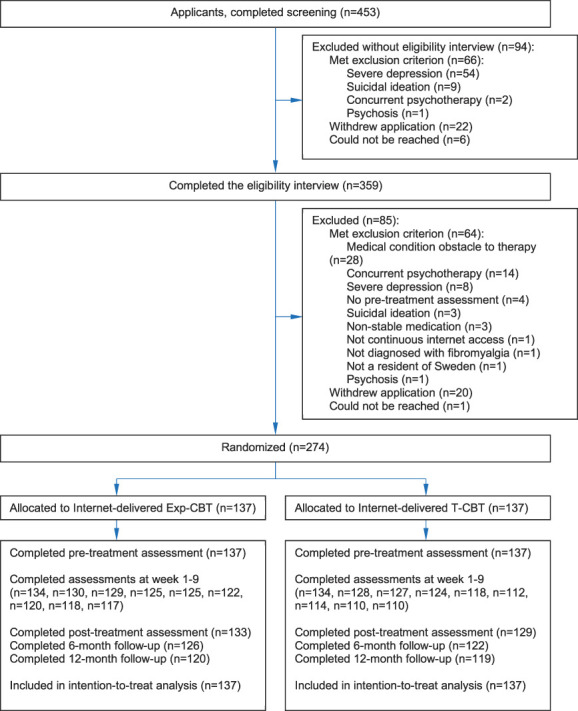
Participant flow and data collection throughout the study period. Exp-CBT, internet-delivered exposure-based cognitive behavior therapy; T-CBT, internet-delivered traditional cognitive behavior therapy.

Recruitment started on September 22, 2021, enrollment was completed on February 16, 2022, and the last follow-up was completed on May 30, 2023 (Fig. 1). The sample consisted of 274 participants who were randomly assigned to Exp-CBT (n = 137) or T-CBT (n = 137). The typical participant was a 51-year-old woman who had lived with the fibromyalgia diagnosis for 11 years and reported moderate to severe symptoms (Table 1).

**Table 1:** Baseline characteristics of participants randomized to internet-delivered exposure-based cognitive behavior therapy or internet-delivered traditional cognitive behavior therapy for fibromyalgia.

	Exp-CBT (n = 137)	T-CBT (n = 137)	Total (N = 274)
Sociodemographic variables			
Mean (SD) age in years, range	50 (12), 21-76	52 (11), 18-76	51 (11), 18-76
Women	135 (99%)	134 (98%)	269 (98%)
Educational attainment			
Compulsory school (≤9 y)	9 (7%)	10 (7%)	19 (7%)
Upper secondary school (≤3 y)	39 (28%)	41 (30%)	80 (29%)
Postsecondary or university	89 (65%)	86 (63%)	175 (64%)
Married or de facto	103 (75%)	103 (75%)	206 (75%)
Occupational status			
Working full or part time	83 (61%)	86 (63%)	169 (62%)
Retired	23 (17%)	22 (16%)	45 (16%)
Disability pension	8 (6%)	11 (8%)	18 (7%)
Unemployed	7 (5%)	7 (5%)	14 (5%)
Student	6 (4%)	7 (5%)	13 (5%)
On sick leave	6 (4%)	2 (1%)	8 (3%)
Other or unclear	4 (3%)	3 (2%)	7 (3%)
Clinical variables			
Fibromyalgia			
Mean (SD) years with diagnosis, range	10 (9), 0-44	11 (9), 0-35	11 (9), 0-44
Overall symptom severity			
Mild (FIQ < 39)	15 (11%)	8 (6%)	23 (8%)
Moderate (39 ≤ FIQ<59)	57 (42%)	55 (40%)	112 (41%)
Severe (FIQ ≥ 59)	65 (47%)	74 (54%)	139 (51%)
Mean (SD) pain catastrophizing (PCS)	20.4 (10.0)	20.2 (9.8)	20.3 (9.8)
Mean (SD) pain avoidance (PIPS-A)	33.1 (8.9)	32.9 (8.2)	33.0 (8.5)
Clinically significant anxiety (GAD-2 ≥ 3)	42 (31%)	50 (36%)	92 (34%)
Clinically significant depression (PHQ-2 ≥ 3)	39 (28%)	53 (39%)	92 (34%)
At least 1 comorbid somatic condition*	94 (69%)	103 (75%)	197 (72%)
Physical activity: insufficiently active (GSLTPAQ)	106 (77%)	109 (80%)	215 (78%)
Mean (SD) functional impairment (WD2-12)	39.8 (16.7)	39.1 (14.0)	39.4 (15.4)

* As reported by the patient in response to the questionnaire item “Do you have any physical illness in addition to fibromyalgia?”

Exp-CBT, internet-delivered exposure-based cognitive behavior therapy; FIQ, Fibromyalgia Impact Questionnaire, scored 0-100; GAD-2, 2-item Generalized Anxiety Disorder, scored 0-6; GSLTPAQ, Godin-Shephard Leisure-Time Physical Activity Questionnaire, “insufficiently active” implies <24; PCS, Pain Catastrophizing Scale, scored 0-52; PHQ-2, 2-item Patient Health Questionnaire, scored 0-6; PIPS-A, Psychological Inflexibility in Pain Scale-Avoidance subscale, scored 8-56; postsecondary or university, International Standard Classification of Education (ISCED) 1997 level 4 or higher; T-CBT, internet-delivered traditional cognitive behavior therapy; WD2-12, 12-item World Health Organization Disability Assessment Schedule 2, scored 0-100.

### 3.2. Missing data points

All participants completed the pretreatment assessment, and 2734 of 3014 (91%) weekly FIQ scores were collected over the 11 assessment points of the treatment period (Fig. 1). The posttreatment assessment was completed by 133 of 137 (97%) in Exp-CBT vs 129 of 137 (94%) in T-CBT.

### 3.3. Primary outcome and potential moderators

**Figure 2.**
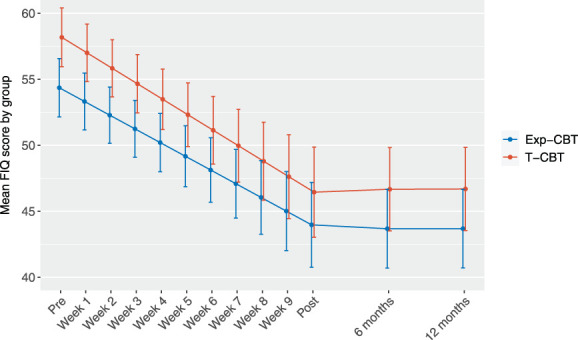
Change in fibromyalgia severity from pretreatment to 12 months after treatment termination. Exp-CBT, internet-delivered exposure-based cognitive behavior therapy; FIQ, Fibromyalgia Impact Questionnaire; T-CBT, internet-delivered traditional cognitive behavior therapy.

There was no significant difference between Exp-CBT and T-CBT in the mean reduction of overall fibromyalgia severity from pretreatment to posttreatment (b = 1.3, 95% CI −3.0 to 5.7, *P* = 0.544, d = −0.10; Fig. 2, Table 2). Although this reduction in overall fibromyalgia severity was larger for patients with higher baseline severity in both treatments, the relative effect of Exp-CBT and T-CBT was not moderated by baseline age, educational attainment, years with the diagnosis, overall severity, pain intensity, pain avoidance behaviors, or pain catastrophizing (seeing that fibromyalgia has a much lower prevalence in men than in women, we anticipated a very small number of male participants and did not analyze gender or sex as a potential moderator). However, in a nonplanned moderator analysis suggested by a reviewer, baseline physical activity did moderate the outcome such that a higher level of physical activity (active vs insufficiently active) was predictive of a 6.8 point larger reduction in fibromyalgia severity (the FIQ) in T-CBT, but not in Exp-CBT (Table SDC2, available at http://links.lww.com/PAIN/B966). As can be seen in Figure 2 and Table 2, effects were maintained up to 12 months after treatment.

**Table 2:** Efficacy of internet-delivered exposure-based cognitive behavior therapy vs internet-delivered traditional cognitive behavior therapy for fibromyalgia.

		Estimated means			Change over pre–post main phase (to the primary end point)							Pre-12 MFU, ie, including the follow-up phase					
		Pre	Post	12 MFU	Within-group			Between-group difference				Within-group change			Between-group difference		
					b	95% CI	*d*	b	95% CI	*d*	*P*	b	95% CI	*d*	b	95% CI	*d*
Primary outcome																	
Fibromyalgia severity FIQ (0-100)	Exp-CBT T-CBT	54.4 58.2	44.0 46.4	43.7 46.7	−10.4 −11.7	−13.4 to −7.4 −14.9 to −8.6	−0.77 −0.87	1.3	−3.0 to 5.7	−0.10	0.544	−10.9 −11.6	−13.6 to −8.2 −14.4 to −8.8	−0.81 −0.86	0.7	−3.2 to 4.6	−0.05
Secondary outcomes																	
Pain intensity, primary FIQ-pain (0-10)	Exp-CBT T-CBT	5.7 6.2	4.9 5.4	4.9 5.4	−0.8 −0.7	−1.2 to −0.4 −1.1 to −0.3	−0.41 −0.38	0.0	−0.6 to 0.5	0.02	0.883	−0.8 −0.8	−1.2 to −0.5 −1.1 to −0.4	−0.44 −0.41	−0.1	−0.6 to 0.5	0.03
Pain intensity BPI-SF (0-10)	Exp-CBT T-CBT	4.8 5.1	3.9 4.4	3.6 4.2	−0.8 −0.7	−1.3 to −0.4 −1.1 to −0.3	−0.43 −0.38	−0.1	−0.7 to 0.5	0.06	0.721	−1.2 −1.0	−1.6 to −0.7 −1.4 to −0.5	−0.61 −0.51	−0.2	−0.8 to 0.4	0.10
Fatigue FSS (9-63)	Exp-CBT T-CBT	49.1 49.8	42.0 41.1	42.6 43.5	−7.1 −8.7	−9.0 to −5.3 −10.6 to −6.8	−0.73 −0.90	1.6	−1.1 to 4.3	−0.16	0.243	−6.5 −6.3	−8.4 to −4.6 −8.3 to −4.4	−0.67 −0.65	−0.2	−3.0 to 2.6	0.02
Depression PHQ-2 (0-6)	Exp-CBT T-CBT	2.0 2.3	1.6 1.5	1.6 1.7	−0.4 −0.9	−0.7 to −0.1 −1.2 to −0.6	−0.25 −0.53	0.5	0.0 to 0.9	−0.28	0.030*	−0.3 −0.7	−0.6 to 0.0† −1.0 to −0.4	−0.20 −0.41	0.4	−0.1 to 0.8	−0.22
General anxiety GAD-2 (0-6)	Exp-CBT T-CBT	1.9 2.1	1.5 1.6	1.6 1.7	−0.4 −0.5	−0.7 to −0.1 −0.8 to −0.2	−0.22 −0.30	0.1	−0.3 to 0.5	−0.08	0.484	−0.4 −0.4	−0.7 to −0.1 −0.7 to −0.1	−0.21 −0.24	0.0	−0.4 to 0.5	−0.03
Pain catastrophizing PCS (0-52)	Exp-CBT T-CBT	19.0 19.3	11.4 12.4	11.7 12.7	−7.6 −6.9	−9.0 to −6.1 −8.4 to −5.4	−0.77 −0.70	−0.6	−2.7 to 1.5	0.07	0.547	−7.3 −6.6	−8.7 to −5.9 −8.1 to −5.2	−0.74 −0.67	−0.7	−2.7 to 1.3	0.07
Disability WD2-12 (0-100)	Exp-CBT T-CBT	39.8 39.1	29.5 32.4	28.8 33.3	−10.2 −6.8	−12.7 to −7.8 −9.2 to −4.3	−0.67 −0.44	−3.5	−6.9 to 0.0	0.23	0.047*	−10.9 −5.8	−13.6 to −8.2 −8.3 to −3.4	−0.71 −0.38	−5.1	−8.8 to −1.4	0.33
Quality of life BBQ (0-96)	Exp-CBT T-CBT	45.6 47.8	57.4 55.3	55.8 53.7	11.7 7.5	8.6 to 14.9 4.5 to 10.5	0.57 0.36	4.2	−0.1 to 8.6	−0.20	0.057	10.1 5.9	6.7 to 13.5 2.4 to 9.5	0.49 0.29	4.2	−0.6 to 9.0	−0.20

Linear mixed effects regression models fitted on multiply imputed data in accordance with the intention-to-treat principle (N = 274). The primary analysis of the pretreatment to posttreatment main phase was conducted by an individual blind to treatment identity, before the completion of the follow-up phase, which was then imputed using a second, separate, multiple imputation procedure that also incorporated previous measurement points. Between-group effects refer to the group different in mean change (slope). The FIQ, FIQ-pain, and PCS were administered on a weekly basis over the main phase, which was modeled over 11 measurement points. The BPI-SF, FSS, PHQ-2, GAD-2, WD2-12, and BBQ were administered before and after treatment, and the main phase was modeled over 2 measurement points.

* Statistically significant at α = 0.05.

† The upper bound of this confidence interval was below zero, and the coefficient was thus statistically significant (*P* < 0.05).

12MFU, 12-month follow-up, approximately 12 months after the posttreatment assessment; BBQ, Brunnsviken Brief Quality of Life, scored 0-96; BPI-SF, Brief Pain Inventory-Short Form, Severity subscale, scored 0-10; Exp-CBT, internet-delivered exposure-based cognitive behavior therapy; FIQ, Fibromyalgia Impact Questionnaire, scored 0-100; FIQ-pain, Fibromyalgia Impact Questionnaire-Pain subscale, scored 0-10; FSS, Fatigue Severity Scale, scored 9-63; GAD-2, 2-item Generalized Anxiety Disorder, scored 0-6; PCS, Pain Catastrophizing Scale, scored 0-52; PHQ-2, 2-item Patient Health Questionnaire, scored 0-6; T-CBT, internet-delivered traditional cognitive behavior therapy; WD2-12, 12-item World Health Organization Disability Assessment Schedule 2, scored 0-100.

### 3.4. Credibility, satisfaction, and secondary efficacy outcomes

Treatment credibility and the expectancy of improvement was rated about as high in Exp-CBT as in T-CBT (M = 32.1, SD = 10.7, n = 135 vs M = 34.1, SD = 9.7, n = 134). Although there was a significantly smaller mean reduction in depression in Exp-CBT as compared to T-CBT (b = 0.5, *P* = 0.030, d = −0.28), the participants in Exp-CBT reported a larger mean reduction in disability (b = −3.5, *P* = 0.047, d = 0.23). The difference in effect on depression was no longer significant at 12-month follow-up, whereas the difference in disability was slightly larger (b = −5.1, d = 0.33). There was no significant Exp-CBT vs T-CBT difference in the effect on pain intensity, fatigue, general anxiety, catastrophizing, or quality of life (Table 2). Minimal clinically important improvement was seen in 60% (80/133) in Exp-CBT vs 59% (76/129) in T-CBT (valid RR = 1.02, χ^2^ = 0.04, *P* = 0.839). Minimally clinically important deterioration was seen in 17% (22/133) in Exp-CBT vs 12% (16/129) in T-CBT (valid RR = 1.33, χ^2^ = 0.90, *P* = 0.342). Participant satisfaction was adequate and did not differ significantly between Exp-CBT and T-CBT (M = 25.4, SD = 5.4, n = 131 vs M = 26.5, SD = 5.1, n = 129; t = −1.65, *P* = 0.100). More secondary efficacy outcomes are found in the Supplemental digital content (available at http://links.lww.com/PAIN/B966).

### 3.5. Blinding and adherence to the study protocol

Three participants in Exp-CBT (2%) and 1 participant in T-CBT (1%) were possibly unblinded to study design, but no participants were unblinded to the superiority hypothesis (see supplemental digital content, available at http://links.lww.com/PAIN/B966). Variables pertaining to adherence are reported in Table 3. There was no significant difference between Exp-CBT and T-CBT in completer or dropout rates. Although therapists spent slightly more time on phone calls and messages in Exp-CBT, there was little difference in the overall time spent per patient and week (M = 20.2 minutes, SD = 20.2 vs M = 17.1 minutes, SD = 12.5). Patients identified less with the treatment model in Exp-CBT than in T-CBT but were more likely to decrease or end their pain medication in Exp-CBT than in T-CBT.

**Table 3:** Integrity of the study protocol.

	Exp-CBT (n = 137)	T-CBT (n = 137)	Difference
Module completion and drop-outs			
Completed treatment (≥5 modules initiated)	95/137 (69%)	97/137 (71%)	RR = 0.98; χ^2^ = 0.07, *P* = 0.792
Mean (SD) modules initiated out of 8, range	5.8 (2.4), 1-8	6.0 (2.5), 1-8	
Dropped out (≥3 wk inactivity or explicit)	23/137 (17%)	32/137 (23%)	RR = 0.72; χ^2^ = 1.84, *P* = 0.175
Main phase patient engagement			
Mean (SD) patient h/wk, range	6.7 (5.7), 0-30, n = 122	6.7 (5.9), 0-42, n = 122	t = 0.00, df = 242, *P* = 1.000
Mean (SD) identification (0-4) with treatment model, range	2.7 (1.2), 0-4, n = 115	3.1 (0.9), 0-4, n = 116	t = 2.76, df = 229, *P* = 0.006*
Mean (SD) exposure exercises/reported week, range	5.5 (4.1), 0-27.4, n = 116	Not applicable	
Mean (SD) mindfulness exercises/reported week, range	13.3 (9.5), 0-54.6, n = 136	Not applicable	
Mean (SD) relaxation exercises/reported week, range	Not applicable	7.7 (5.4), 0-29.1, n = 135	
Interaction with the therapist			
Mean (SD) strength of relationship (WAI)	69.6 (13.7), 24-84, n = 135	70.4 (13.6), 23-84, n = 134	t = 0.46, df = 267, *P* = 0.646
Mean (SD) total therapist minutes, range	202.5 (201.9), 4.1-1239.9	171.3 (124.7), 1.6-925.2	t = −1.54, df = 226.6†, *P* = 0.125
Mean (SD) total number of phone calls, range	1.0 (1.2), 0-6	1.0 (1.5), 0-7	
Mean (SD) total phone calls minutes, range	19.1 (34.5), 0-230	9.0 (19.9), 0-150	t = −2.94, df = 217.8†, *P* = 0.004*
Mean (SD) total number of participant messages, range	15.7 (13.0), 0-100	12.2 (8.8), 0-52	t = −2.65, df = 238.3†, *P* = 0.009*
Mean (SD) total number of therapist messages, range	23.6 (9.5), 3-72	21.2 (8.3), 1-42	t = −2.25, df = 272, *P* = 0.025*
Enrollment in other treatments			
New or increased psychotropic medication	4/131 (3%)	3/129 (2%)	RR = 1.31; χ^2^ = 0.13, *P* = 0.717
Stopped or decreased psychotropic medication	4/131 (3%)	1/129 (1%)	RR = 3.94; χ^2^ = 1.79, *P* = 0.181
New or increased pain medication	10/131 (8%)	10/129 (8%)	RR = 0.98; χ^2^ = 0.00, *P* = 0.971
Stopped or decreased pain medication	33/131 (25%)	17/129 (13%)	RR = 1.91; χ^2^ = 6.04, *P* = 0.014*
Met with psychologist or psychotherapist	23/131 (18%)	22/129 (17%)	RR = 1.03; χ^2^ = 0.01, *P* = 0.915
Met with general practitioner	48/131 (37%)	50/129 (39%)	RR = 0.95 χ^2^ = 0.12, *P* = 0.725
Met with specialist physician	27/131 (21%)	30/129 (23%)	RR = 0.89 χ^2^ = 0.27, *P* = 0.606

Tests of continuous and count variables focused on arithmetic means in accordance with the preregistered statistical analysis plan. Due to conceptual overlaps with other variables, an a priori decision was made not to test the number of modules (over completers) or the number of phone calls (over time spent on phone calls).

* Statistically significant at α = 0.

† Due to unequal group variances, we used the Welch-Satterthwaite approximation of the degrees of freedom for these tests.

Exp-CBT, internet-delivered exposure-based cognitive behavior therapy; T-CBT, internet-delivered traditional cognitive behavior therapy; WAI, brief version of the Working Alliance Inventory, scored 12-84.

### 3.6. Adverse events

No serious adverse event was reported and deemed to be related to treatment or participation in the trial. Four participants, all in T-CBT, reported experiencing a serious medical condition warranting hospitalization. Two participants had a serious COVID-19 infection, 1 had a suspected tumor, and 1 had a suspected stroke. At least 1 adverse event was reported by 26% (34/131) of the participants in Exp-CBT vs 22% (28/129) in T-CBT. This difference was not statistically significant (RR = 1.20; χ^2^ = 0.65, *P* = 0.422).

## 4. Discussion

Unexpectedly, this well-powered randomized trial found that Exp-CBT was not superior to T-CBT in reducing fibromyalgia severity over the 10-week treatment period. Both therapies had moderate to large within-group effects that were maintained up to 12 months after treatment. Exp-CBT had a somewhat larger effect on disability, a difference that was larger at 12-month follow-up (d = 0.33). T-CBT had a somewhat larger effect on symptoms of depression, perhaps because it involves behavioral activation that is a reference standard treatment for depression,^2^ although this difference was no longer significant at 12-month follow-up. Moderator analyses revealed that the relative effect of Exp-CBT vs T-CBT was not moderated by baseline age, educational attainment, years with the diagnosis, overall severity, pain intensity, pain avoidance behaviors, or pain catastrophizing. In a nonplanned moderator analysis prompted by the peer-reviewing process, we found that participants did better in T-CBT, but not Exp-CBT, if they had a higher level of physical activity at baseline. On the whole, the 2 treatments produced relatively similar effects.

To the best of our knowledge, this is the second largest psychological treatment trial for fibromyalgia^26,37^ and one of the first to compare 2 active bona fide treatments.^7^ We used a rigorous control condition considering that the T-CBT protocol^10^ has been in use at a tertiary pain clinic for approximately 10 years. Moreover, the 2-site design enhanced allegiance and adherence to both treatments, eligibility criteria allowed for psychiatric and somatic comorbidity, and moderator analyses were in line with expert recommendations.^7^ Thus, several methodological strengths speak for the validity of this trial.

The results ran contrary to our hypothesis of Exp-CBT superiority over T-CBT and were unexpected in light of a recent movement within the field as a whole toward an increased preference for novel variants of cognitive behavior therapy, not least so called “third-wave” treatments. For example, a recent editorial concluded that *“the evolution of CBT in different waves has allowed more effective treatments to be developed and to be made available to patients with chronic pain in various forms*.*”*^29^ The fact that Exp-CBT and T-CBT had largely similar effects in fibromyalgia highlights the continued need for direct comparisons of nonpharmacological treatments in adequately powered high-quality trials.

The within-group effect of Exp-CBT in the present trial was smaller than that seen in a previous RCT, as evidenced by a mean FIQ reduction of 10.4 (d = 0.77) as compared to 16.9 (d = 1.04) using equivalent statistical models.^19^ One possible explanation could be that therapists were instructed to reply within 48 hours and on weekdays only, as opposed to 24 hours regardless of the day in the previous trial. In addition, the present trial advertised on social media platforms to a larger extent, whereas the previous trial relied more on recruitment through newspapers and patient organizations. Although this might have attracted slightly different populations, we note that there were no clear differences between the trials in baseline symptoms or the number of initiated modules. Yet another potential explanation could be the COVID-19 pandemic, which may have led to increased distress and lowered motivation for exposure. Several participants also reported contracting COVID-19 during the trial, which may have affected symptoms and adherence.^57^

No previous RCT has compared Exp-CBT with another active treatment for fibromyalgia. Boersma et al.^8^ compared a face-to-face hybrid treatment integrating exposure and emotion regulation strategies with internet-delivered T-CBT for adults with chronic musculoskeletal pain and comorbid symptoms of anxiety or depression (N = 115). Results were similar to that of this study in that the exposure-based treatment had larger mean effects than T-CBT on disability (“interference”: d = 0.51-0.63), whereas there was no such apparent advantage in effects on anxiety (d = 0.06-0.26) or pain intensity (−0.26 to 0.10). There were, however, also differences, primarily in that we saw no added effect of Exp-CBT over T-CBT on pain catastrophizing (d = 0.07 vs Boersma et al.'s d = 0.27-0.39), and also that T-CBT (not Exp-CBT) had a superior effect on depression (d = −0.28 vs Boersma et al.'s d = 0.37-0.43). In another RCT by Glombiewski et al.,^16^ Exp-CBT was compared with T-CBT for patients with chronic back pain (N = 88). The treatments did not differ significantly in their mean effects on pain intensity (d = −0.09 to 0.03), which is similar to this study, or pain disability (d = −0.01 to 0.15), which is different from this study.

On the whole, our findings pertaining to fibromyalgia show some resemblance to research on other chronic pain conditions in that pain intensity may respond about equally well to Exp-CBT and T-CBT, although there are also differences between trials for example in the relative effect on disability and depression. These differences can probably be attributed to the precise content of the T-CBT protocol and also the participants' primary diagnosis. For example, although our T-CBT protocol included behavioral activation, this was not the case in the study by Boersma et al. who saw very different effects on depression. In this trial, both Exp-CBT and T-CBT produced clinically relevant effects and led to adequate levels of participant satisfaction. Notably, in contrast with mainstream theoretical work,^52^ there was also no indication in the moderator analyses (Table SDC2, available at http://links.lww.com/PAIN/B966) that the level of baseline behavioral avoidance, which is the main target of Exp-CBT, determined whether Exp-CBT or T-CBT was more effective.

The results from this trial should be interpreted in light of its limitations. There was no passive control group, which means that we cannot infer from this trial alone that improvement had not been seen without a clinical intervention. This said, we found Exp-CBT to be superior to a waitlist in a previous RCT for fibromyalgia (d = 0.90). In that trial, symptoms were unlikely to remit spontaneously, as evidenced by an increase in the waitlist FIQ mean of 2.3 (d = −0.14) over 10 weeks.^19^ Similarly, because the present trial did not include a comparator that controlled for nonspecific effects such as the attention of a caregiver and engagement with a structured protocol, we cannot estimate the role of nonspecific effects. Another limitation is that although activity on the platform could be observed, and participant self-reports were indicative of adequate adherence, certain key aspects of the patient's behavior such as how exposure and relaxation exercises were conducted were not directly observed. Moreover, the present trial was not conducted in a healthcare setting, and it was therefore necessary to exclude participants with certain comorbidities, notably severe depression or suicidal ideation. Furthermore, self-referral could suggest high levels of motivation and digital literacy, and educational attainment was high, which limits generalizability to certain contexts. Although it would be expected that dropout rates are higher with other sampling strategies, it should also be pointed out that smaller effects of similar therapies have not always been seen when participants are referred from routine care.^3,44^ Although all participants reported having received a fibromyalgia diagnosis by a physician, the fact that no formal assessment of diagnostic criteria was conducted at the intake interview means that some participants may not have met full diagnostic criteria at the time of the study. Although this can be regarded as a methodological limitation, this is also similar to routine care where patients may long identify with the fibromyalgia diagnosis despite no longer meeting full criteria. Moreover, fibromyalgia severity was moderate to severe in this sample (Table 1), which is similar to many routine care settings.^7^

The outcome of this trial contributes to nuancing the discussion within psychological treatment research for chronic pain. In recent years, high-quality trials evaluating T-CBT have been few in comparison to the inflow of studies evaluating “third-wave” therapies.^17,49^ Still, results from the current trial suggest that T-CBT can stand its ground as a reference standard intervention for fibromyalgia, with moderate to large effects on overall severity and fatigue, clearly highlighting the value of further implementation. Tentatively, based on current evidence, both treatments evaluated in this trial seem to be viable, and the choice of treatment may come down to the preference of the patient and individual caregiver. We hope that the results from the current trial will serve as a motivator to the research community in conducting rigorous head-to-head comparisons of existing credible treatments, including T-CBT, with the overarching goal of gaining more knowledge about what works best for whom and under what circumstances.

Our results warrant replication in future trials. Further research is also needed to disentangle the mechanisms of Exp-CBT and T-CBT, to determine the specific contribution of treatment components to the overall treatment effect, and to identify better methods of predicting the treatment response. Future publications are planned on cost-effectiveness and mediators of treatment effects.

In conclusion, internet-delivered exposure-based cognitive behavior therapy (Exp-CBT) does not seem to be superior to internet-delivered traditional cognitive behavior therapy (T-CBT) for fibromyalgia. In this trial, both treatments were associated with a marked reduction in fibromyalgia severity, which means that the online treatment format might be of high clinical utility. T-CBT can still be regarded a reference standard treatment for fibromyalgia that is likely to stand up relatively well to more recently developed therapies.

## Conflict of interest statement

E.H-L., B.Lj., and E.A. have co-authored a self-help book for pathological health anxiety that is based on exposure-based cognitive behavior therapy and for which they receive royalties. E.H-L. and B.Lj. are shareholders of Hedman-Lagerlöf och Ljótsson Psykologi AB which licences exposure-based cognitive behavior therapy for irritable bowel syndrome.

## Appendix A. Supplemental digital content

Supplemental digital content associated with this article can be found online at http://links.lww.com/PAIN/B966.

## Supplementary Material
