# Supplementary material for: Effect of exposure-based vs traditional cognitive behavior therapy for fibromyalgia: a two-site single-blind randomized controlled trial
Source: Pain. 2023 Dec 15;165(6):1278–88. doi: 10.1097/j.pain.0000000000003128 (PMC11090030; doi:10.1097/j.pain.0000000000003128)
Supplement: Supplementary file 1 [file jop-165-1278-s001.pdf]

Supplementary digital content ("Effect of exposure-based versus traditional cognitive behavior therapy for fibromyalgia: A two-site single-blind randomized controlled trial")

Supplementary digital content

Table SDC1. Overview of treatment protocols 2

Table SDC2. Tests of potential baseline moderators of the between-group difference in the pre- to post-treatment effect of Internet-delivered exposure-based cognitive behavior therapy (Exp-CBT) versus Internet-delivered traditional cognitive behavior therapy (T-CBT) for fibromyalgia 7

Minimal clinically important improvement/deterioration 9

Reliable improvement/deterioration on an individual basis 10

Post-treatment assessment of blinding 11

References..13

Supplementary digital content ("Effect of exposure-based versus traditional cognitive behavior therapy for fibromyalgia: A two-site single-blind randomized controlled trial")

Table SDC1. Overview of treatment protocols

Module	Exp-CBT	T-CBT
1	Introduction to the treatment and fibromyalgia, focusing on the role of behaviors and mindfulness<br><br>Content: Introduction to the treatment format, the online platform, and fibromyalgia. Overview of common problems in fibromyalgia, and the role of physiological processes, cognitions, emotions, and behaviors in the modulating the experience of pain and pain-related distress. The role of avoidance and safety behaviors in exacerbating symptoms in the long term. Registering and creating an overview of own avoidance and safety behaviors in relation to situations, introducing the daily behavior diary. Practicing mindfulness as a standalone exercise and in everyday life.<br><br>Key worksheets/fact sheets*:<br>• Behavior diary for daily registration<br>• My avoidance and safety behaviors<br>• Introduction to mindfulness exercises	Introduction to the treatment and fibromyalgia, focusing on a multidimensional biopsychosocial model of fibromyalgia<br><br>Content: Introduction to the treatment format, the online platform, and fibromyalgia. Overview of common problems in fibromyalgia, and an introduction to the multidimensional biopsychosocial model of fibromyalgia. The role of certain key processes such as muscle tension and social withdrawal in exacerbating symptoms. On the importance of evaluating various strategies to address the multidimensional condition of fibromyalgia. Setting meaningful goals for the treatment, identifying difficult situations where strategies are needed, and practicing relaxation.<br><br>Key worksheets/fact sheets*:<br>• Goals for the treatment<br>• Planning strategies to manage challenging situations<br>• Introduction to relaxation<br>• Logbook for relaxation
2	Behaviors in fibromyalgia<br><br>Content: On the interaction between perception/hypervigilance and avoidance and safety behaviors. The benefits of not acting on pain and related discomfort. Practicing mindfulness and response prevention to reduce pain, pain-related discomfort, and disability in the long term.<br><br>Key worksheets/fact sheets*:<br>• Behavior diary for daily registration<br>• My avoidance and safety behaviors<br>• Introduction to mindfulness exercises<br>• Practicing mindfulness in the presence of symptoms	Promotion of physical exercise<br><br>Content: Beneficial effects of physical exercise. Ready-made exercises for increased stability and stretching. Making a realistic plan for physical exercise.<br><br>Key worksheets/fact sheets*:<br>• Planning strategies to manage challenging situations<br>• Relaxation, step 2<br>• Logbook for relaxation<br>• Plan for physical exercise
3	Thoughts and emotions in fibromyalgia	Activity scheduling and pacing techniques

	Content: On the impact of negative automatic thoughts on emotional and behavioral responses. The benefits of identifying thoughts as thoughts, and emotional responses as emotional responses, without the need to act on the impulse to reduce discomfort in the short term.<br><br>Key worksheets/fact sheets*:<br>• Behavior diary for daily registration<br>• Common avoidance and safety behaviors<br>• Practicing mindfulness also in the presence of symptoms<br>• Practicing exposure in the presence of negative thoughts<br>• "Sitting still": practicing observing physical sensations	Content: On the importance of pacing, i.e., managing activity level in the short term, often in response to symptoms. Planning and taking pauses, being mindful of pain levels, planning for recuperation, and maintaining a consistent and manageable level of activity. Planning activities including the use of strategies such as pacing, resting, and physical exercise. Use of strategies before, during, and after activities.<br><br>Key worksheets/fact sheets*:<br>• Planning strategies to manage challenging situations<br>• Relaxation, step 3<br>• Logbook for relaxation<br>• Plan for physical exercise<br>• Activity scheduling
4	Introduction to exposure in vivo<br><br>Content: Systematic repeated exposure as a means of reducing the impact of pain and pain-related discomfort, and learning new ways of managing difficult situations. Hands-on examples of exposure exercises. Optimizing the effects of exposure: planning, starting small if necessary, staying in the situation, sticking with response prevention, and adopting an accepting stance toward thoughts, emotions, and physical sensations.<br><br>Key worksheets/fact sheets*:<br>• "My challenges": avenues for exposure in vivo<br>• Systematic evaluation of exposure and response prevention<br>• Practicing mindfulness also in the presence of symptoms<br>• Practicing exposure in the presence of negative thoughts<br>• "Sitting still": practicing observing physical sensations	Addressing negative thoughts in fibromyalgia<br><br>Content: Introduction to cognitive restructuring techniques: examining the evidence, evaluating alternative interpretations, evaluating effects of cognitions. Alternatively, being accepting of thoughts. Common cognitive distortions: catastrophizing, black-and-white thinking, should and ought, mind-reading and jumping to conclusions, and over-generalizing.<br><br>Key worksheets/fact sheets*:<br>• Planning strategies to manage challenging situations<br>• Relaxation, step 4<br>• Plan for physical exercise<br>• Activity scheduling<br>• Challenging negative thoughts
5	Tricks of the trade and problem-solving exposure<br><br>Content: The rest of the treatment will continue to focus on practicing exposure and mindfulness. Benefits of planning exposure in advance.	Acceptance and mindfulness techniques<br><br>Content: On the impossibility of maintaining control of one's thoughts. The benefits of identifying thoughts as thoughts. Brief exercise: observing

	Solutions to common obstacles: forgetting about exposure, choosing what to work with, overcoming resistance to exposure, preventing the premature termination of exposure exercises, implications of high levels of distress, identifying the appropriate level of difficulty, finding the time for exposure, managing setbacks and successfully resuming treatment.<br><br>Key worksheets/fact sheets*:<br>• "My challenges": avenues for exposure in vivo<br>• Systematic evaluation of exposure and response prevention<br>• Scheduling exposure<br>• Practicing mindfulness also in the presence of symptoms<br>• "Sitting still": practicing observing physical sensations	thoughts in an objective manner. On the benefits of distinguishing between that which can be changed, and that which has to be accepted.<br><br>Key worksheets/fact sheets*:<br>• Planning strategies to manage challenging situations<br>• Relaxation, step 4<br>• Plan for physical exercise<br>• Activity scheduling<br>• Being present in the moment
6	Continued exposure<br><br>Content: A fictitious example (case) of an individual with fibromyalgia working continuously with the treatment strategies, managing obstacles, and obtaining beneficial long-term effects. Understanding worry as the "ping pong game" of discomfort associated with physical sensations and negative automatic thoughts on the one hand, and verbal behavior with the aim of reducing discomfort on the other hand. On the importance of keeping with the core treatment principles including planned exposure exercises, continuous response prevention, and practicing mindfulness both as part of standalone exercises and as part of everyday life. How to set goals for the rest of the treatment.<br><br>Key worksheets/fact sheets*:<br>• "My challenges": avenues for exposure in vivo<br>• Systematic evaluation of exposure and response prevention<br>• Scheduling exposure<br>• Practicing mindfulness also in the presence of symptoms<br>• "Sitting still": practicing observing physical sensations	Stress and stress-reduction techniques<br><br>Content: What is stress? Brief conceptual, evolutionary, and physiological introduction. On the acute stress response, and the fact that experiences and biology shape its triggers. Introduction to stress-reduction: focusing on what one ones to achieve, maintaining an objective view of the world (addressing thoughts by means of cognitive restructuring or acceptance), evaluating whether treats are real (for example by trying new behaviors), increasing resistance (for example by problem-solving), recuperation and relaxation. Introduction to assertiveness and setting boundaries: saying no and giving feedback.<br><br>Key worksheets/fact sheets*:<br>• Planning strategies to manage challenging situations<br>• Relaxation, step 4<br>• Plan for physical exercise<br>• Activity scheduling<br>• Planning to say no
7	Summary and direction in life	Sleep improvement techniques

	Content: Summarizing the treatment. Introduction to values via exercise: Envision looking back at your life as a very old person, and reflecting on what you would like to achieve and stand for. On the importance of striving towards something positive, beyond reducing discomfort. How to find one's own values, and valuable behaviors, in the domains of close relationships, work and education, leisure time and interests, and daily responsibilities.<br><br>Key worksheets/fact sheets*:<br>• Own treatment summary<br>• Values and valuable behaviors<br>• Systematic evaluation of exposure and response prevention<br>• Scheduling exposure<br>• "Sitting still": practicing observing physical sensations	Content: What is sleep? Brief conceptual, evolutionary, and physiological introduction. Information about biological rhythms, and the resilience of systems promoting sleep. Introduction to sleep improvement techniques: sleep hygiene, physical exercise, avoiding daytime naps, waking up the same time, winding down in the evening, variations on stimulus control.<br><br>Key worksheets/fact sheets*:<br>• Planning strategies to manage challenging situations<br>• Relaxation, step 4<br>• Plan for physical exercise<br>• Activity scheduling<br>• Planning to say no
8	Preventing relapse and promoting continued improvement<br><br>Content: Encouragement of progress in practicing exposure and response prevention. Maintaining and building on progress by continued work with the treatment strategies: remembering key information and planning for the future. Managing setbacks by identifying what happened, planning for relevant exposure, conducting exposure, and practicing mindfulness.<br><br>Key worksheets/fact sheets*:<br>• Own treatment summary<br>• Values and valuable behaviors<br>• Systematic evaluation of exposure and response prevention<br>• Scheduling exposure<br>• Planning to prevent a relapse	Preventing relapse and promoting continued improvement<br><br>Content: Encouragement of progress in working with the program and evaluating various strategies to manage fibromyalgia. Maintaining and building on progress by continued work with the treatment strategies: remembering key information and planning for the future. Managing setbacks by identifying what happened, setting goals, and planning for the use of relevant strategies.<br><br>Key worksheets/fact sheets*:<br>• Planning strategies to manage challenging situations<br>• Relaxation, step 4<br>• Plan for physical exercise<br>• Activity scheduling<br>• Planning to prevent a relapse

Both protocols had been evaluated in previous randomized controlled trials,[1 2] were delivered via the same online platform, were about equally long, about equally easy to read, and included a roughly similar number of figures. In both treatments, the therapist granted the patient access to new modules, sequentially and contingent on progress. The first part of module 1 – which concerned the treatment format, the online platform, and general information

about fibromyalgia – was identical in the two protocols. Exp-CBT= Internet-delivered exposure-based cognitive behavior therapy. T-CBT=Internet-delivered traditional cognitive behavior therapy.

* Not all worksheets are listed in this table. The participant was free to access all worksheets from previous modules. This was especially common in T-CBT, where the patient was encouraged to evaluate a large number of strategies for managing various aspects of fibromyalgia, and to continue with those found to be most beneficial.

Supplementary digital content ("Effect of exposure-based versus traditional cognitive behavior therapy for fibromyalgia: A two-site single-blind randomized controlled trial")

Table SDC2. Tests of potential baseline moderators of the between-group difference in the pre- to post-treatment effect of Internet-delivered exposure-based cognitive behavior therapy (Exp-CBT) versus Internet-delivered traditional cognitive behavior therapy (T-CBT) for fibromyalgia

	Baseline score distribution			Within-group moderating effect				Between-group moderating effect			
				Treatment	Time (0-1) × Moderator			Group (Exp-CBT=1 vs. T-CBT=0) × Time (0-1) × Moderator			
**Potential moderator**	Mean/n	SD/%	Range		b	z	P	b (95% CI)	z	Incr. d[a]	P
**Sociodemographics**											
Age	50.9	11.5	18-76	Exp-CBT	-0.22	-1.84	0.066	-0.09 (-0.44 to 0.27)	-0.48	0.01	0.632
				T-CBT	-0.13	-0.96	0.336				
Postsecondary education	175/274	64%	0, 1	Exp-CBT	-3.95	-1.44	0.151	-6.16 (-13.88 to 1.57)	-1.56	0.46	0.118
				T-CBT	2.21	0.78	0.437				
**Clinical variables**											
Years with the diagnosis	10.8	8.6	0-44	Exp-CBT	0.05	0.28	0.779	0.14 (-0.32 to 0.60)	0.61	-0.01	0.542
				T-CBT	-0.10	-0.60	0.546				
Fibromyalgia severity (FIQ)	58.5	13.5	11.8-87.8	Exp-CBT	-0.36	-3.95	<0.001*	-0.04 (-0.32 to 0.24)	-0.29	0.00	0.771
				T-CBT	-0.31	-2.84	0.005*				
Pain intensity (BPI-SF)	4.9	1.9	0-8.5	Exp-CBT	-2.19	-2.94	0.003*	-1.96 (-3.94 to 0.02)	-1.94	0.15	0.052
				T-CBT	-0.23	-0.34	0.736				
Pain avoidance behaviors (PIPS-A)	33.0	8.5	13-55	Exp-CBT	-0.53	-3.57	<0.001*	-0.34 (-0.77 to 0.10)	-1.53	0.03	0.126
				T-CBT	-0.19	-1.17	0.241				
Pain catastrophizing (PCS)	20.3	9.8	0-50	Exp-CBT	-0.21	-1.62	0.106	-0.14 (-0.51 to 0.24)	-0.72	0.01	0.475
				T-CBT	-0.08	0.56	0.573				
Non-planned analysis:											
Sufficient physical activity (GSLTPAQ)	59/274	22%	0, 1	Exp-CBT	1.66	0.67	0.504	6.77 (0.01 to 13.52)	1.96	-0.50	0.050*[b]
				T-CBT	-5.11	-2.14	0.033*				

Linear mixed effects regression models fitted on multiply imputed data in accordance with the intention-to-treat principle (N=274). The non-planned analysis of physical activity as a potential moderator was added during the peer-reviewing process. BPI-SF=Brief Pain Inventory - Short Form, Severity subscale with a theoretical range of 0-10. Exp-CBT=Internet-delivered exposure-based cognitive behavior therapy. FIQ= Fibromyalgia Impact Questionnaire with a theoretical range of 0-100. GSLTPAQ=Godin-Shephard Leisure-Time Physical Activity Questionnaire, dichotomized to indicate sufficient (≥24) vs. insufficient (<24) physical activity. PCS=Pain Catastrophizing Scale with a theoretical range of 0-52. PIPS-A=Psychological Inflexibility in Pain Scale - Avoidance subscale with a theoretical range of 8-56. T-CBT=Internet-delivered traditional cognitive behavior therapy.

Supplementary digital content ("Effect of exposure-based versus traditional cognitive behavior therapy for fibromyalgia: A two-site single-blind randomized controlled trial")

* Statistically significant at α=0.05.

[a] Positive values in this column are indicative of a between-group standardized effect (Cohen's d) more in favor of Exp-CBT over T-CBT at post-treatment for every increase of 1 in the potential baseline moderator. Negative values stand for the opposite, i.e., an implied between-group standardized effect (Cohen's d) that is more in favor of T-CBT over Exp-CBT at post-treatment for every increase of 1 in the potential baseline moderator.

[b] P = 0.04960116.

Supplementary digital content ("Effect of exposure-based versus traditional cognitive behavior therapy for fibromyalgia: A two-site single-blind randomized controlled trial")

Minimal clinically important improvement/deterioration

Minimal clinically important improvement (14% or more on the FIQ): EXP-CBT=80 (valid: 60%) vs. T-CBT=76 (valid: 59%); valid RR=1.02. χ^2=0.041505, df=1, P=0.834; NNT=100

Minimal clinically important deterioration (14% or more on the FIQ): EXP-CBT=22 (valid: 17%) vs. T-CBT=16 (valid: 12%); valid RR=1.33. χ^2=0.90437, df=1, P=0.3416; NNT=20

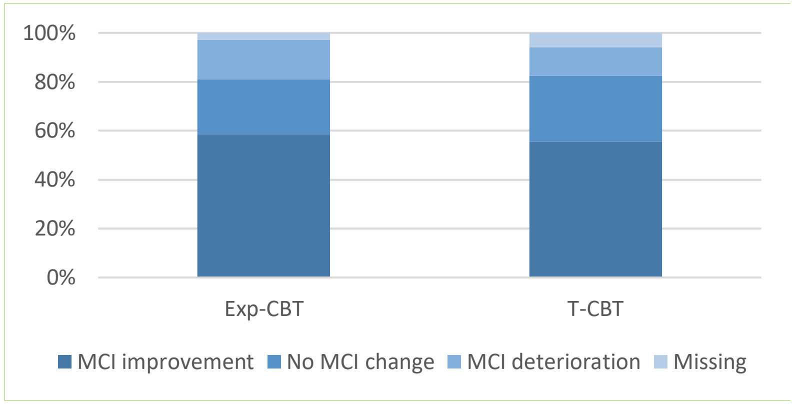

Figure SDC1. Minimal clinically important improvement/deterioration.

Supplementary digital content ("Effect of exposure-based versus traditional cognitive behavior therapy for fibromyalgia: A two-site single-blind randomized controlled trial")

Reliable improvement/deterioration on an individual basis

FIQ S_E=13.51594 × sqrt(1 - 0.81)=5.8914616587
FIQ S_{diff}=sqrt(2 × 5.8914616587^2)=8.33178497993
FIQ RCI criterion=±1.96 × 8.33178497993=±16.3302985607 ≈ ±16.33

Reliable improvement (16.33 or more on the FIQ): EXP-CBT=51 (valid: 38%) vs. T-CBT=54 (valid: 42%); valid RR=0.92. χ^2=0.33683, df=1, P=0.5617; NNT=25

Reliable deterioration (16.33 or more on the FIQ): EXP-CBT=8 (valid: 6%) vs. T-CBT=4 (valid: 3%); valid RR=1.94. χ^2=1.2726, df=1, P=0.2593; NNT=33

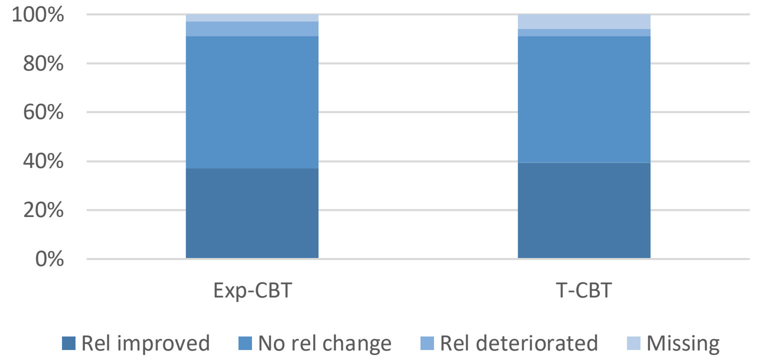

Figure SDC2. Reliable improvement/deterioration on an individual basis.

Supplementary digital content ("Effect of exposure-based versus traditional cognitive behavior therapy for fibromyalgia: A two-site single-blind randomized controlled trial")

Post-treatment assessment of blinding

[Note that because this questionnaire was administered as part of the post-treatment assessment, there was a certain degree of data loss. Answers were available from 131/137 (96%) in Exp-CBT and 129/137 (94%) in T-CBT. Those who answered "No" to question 1 did not complete the following questions.]

In this study, you have been randomized to one of two different treatments.
("I denna studie har du lottats till en av två olika behandlingar.")

1. Do you know which these two treatments were?
("1. Vet du vilka dessa två behandlingar var?")

	Exp-CBT	T-CBT
Yes ("Ja")	6	11
No ("Nej")	89	87
Unsure ("Osäker")	36	31

2. Describe with a few words what these treatments are called or what they amount to:
("2. Beskriv med några få ord vad behandlingarna kallas eller vad de går ut på:")

(If you answered "Unsure" to the question above, explain why and guess which the two treatments were.)
("(Om du svarade "Osäker" på frågan ovan, förklara varför och gissa vilka de två behandlingarna var.)")

	Exp-CBT	T-CBT
Blinded (description not accurate)	39	41
Possibly unblinded (rudimentary description of both treatments)	3	1

3. Do you know with certainty which treatment the researchers believed would be most effective, e.g., because this was revealed during the study?
("3. Vet du med säkerhet vilken behandling forskarna trodde skulle vara mest effektiv, till exempel för att det avslöjades under studien?")

	Blinded according to item 2		Possibly unblinded according to item 2	
	Exp-CBT	T-CBT	Exp-CBT	T-CBT
Yes ("Ja")	6	6	0	0
No ("Nej")	33	35	3	1

[If Yes:] The researchers believe [sic] that the following treatment would be most effective:
("Forskarna tror att följande behandling skulle vara mest effektiv:")

	Blinded according to item 2		Possibly unblinded according to item 2	
	Exp-CBT	T-CBT	Exp-CBT	T-CBT
Reference to exposure	4	0	0	0
No mention of exposure	2	6	0	0

4. Do you know which of these two treatments you were randomized to and enrolled in during the 10-week main phase of the study?
("4. Vet du vilken av dessa två behandlingar du lottades till och gick i under de 10 veckorna av studiens huvudfas?")

	Blinded according to item 2		Possibly unblinded according to item 2	
	Exp-CBT	T-CBT	Exp-CBT	T-CBT
Yes ("Ja")	3*	3	1**	0
No ("Nej")	23	27	0	1
Unsure ("Osäker")	13	11	2	0

* 2/3 answered Yes and made reference to exposure on item 3 (i.e., were possibly aware of the primary study hypothesis).
** This participant answered No on item 3 (i.e., was not aware of the primary study hypothesis).

Supplementary digital content ("Effect of exposure-based versus traditional cognitive behavior therapy for fibromyalgia: A two-site single-blind randomized controlled trial")

References

1. Hedman-Lagerlöf M, Hedman-Lagerlöf E, Axelsson E, et al. Internet-Delivered Exposure Therapy for Fibromyalgia: A Randomized Controlled Trial. *Clin J Pain* 2018;34(6):532-42. doi: 10.1097/AJP.0000000000000566 [published Online First: 2017/10/28]
2. Buhrman M, Syk M, Burvall O, et al. Individualized Guided Internet-delivered Cognitive-Behavior Therapy for Chronic Pain Patients With Comorbid Depression and Anxiety: A Randomized Controlled Trial. *Clin J Pain* 2015;31(6):504-16. doi: 10.1097/AJP.0000000000000176 [published Online First: 2014/11/08]
